# Optimal random search using limited spatial memory

**DOI:** 10.1098/rsos.171057

**Published:** 2018-03-07

**Authors:** Tomoko Sakiyama, Yukio-Pegio Gunji

**Affiliations:** 1 Graduate School of Natural Science and Technology, Okayama University, Okayama 700-8530, Japan; 2 Department of Intermedia Art and Science, School of Fundamental Science and Engineering, Waseda University, Tokyo 169-8555, Japan

**Keywords:** movement strategy, power law, foraging

## Abstract

Lévy walks are known to be efficient movements because Lévy walkers search wide areas while restricting returns to previously visited sites. A self-avoiding walk (SAW) is a series of moves on a lattice that visit the same place only once. As such, SAWs can also be effective search algorithms. However, it is not realistic that foragers memorize many visited positions for a long time. In this work, we investigated whether foragers performed optimal searches when having limited memory. The agent in our model followed SAWs to some extent by memorizing and avoiding visited places. However, the agent lost its memory after a while. In that situation, the agent changed its reactions to visited patches by considering global trail patterns based on local memorized information. As a result, we succeeded in making the agent occasionally produce ballistic walks related to power-law tailed movements across some ranges.

## 1. Introduction

Several studies of animal foraging strategies have reported that Lévy walks are efficient where food is sparse [1]. A Lévy walk is defined as a process whereby an agent takes steps of length *l* at each time and the probability density function of those steps decays asymptotically as a power law

$$P(l)∼l^{-\mu },$$

where 1 < *μ* ≦3.

Lévy walks with the exponent *1* < *μ* < 2 are optimal for a low-density target regime dependent on specific environmental conditions, while optimal Lévy searches with the exponent *μ *∼ 2 are efficient for a broad range of target densities [2,3]. Thanks to scale-free hierarchical movements, Lévy walkers are able to perform wide-range searching while restricting unnecessary returns to previously visited locations [4].

A self-avoiding walk (SAW) consists of moves on a lattice that visit the same place only once [5,6]. As such, SAWs can be applicable to the emergence of Lévy-like movements [7–9]. Animals seem to avoid recently visited places using their spatial memory [10]. However, actual walkers cannot eternally avoid previously visited places because repellent cues such as their memory vanish after a while. Foragers appear to achieve optimal searches using limited information [11–16]. Thus, how do foragers realize effective random walks while avoiding recently visited locations?

Against this background, in this study, we investigated random walks produced by an agent avoiding recently visited places. The agent in our model was allowed to use only recent memory. In general, avoiding visited locations has two meanings for foragers. One is to avoid only that place. The other is to avoid not only that place but also its surroundings. These two meanings are inseparable. To this end, the agent in our model changed its reaction to local visited places. By doing so, we introduced inseparability of the avoidance into our model. As a result, the agent succeeded in producing Lévy-like walks over some ranges.

## 2. Material and methods

Two-dimensional (2D) switching SAW of recently visited locations.

Each trial is run for a maximum of 1000 time steps. We use a 2D SAW of recently visited locations to set the simulation stage for each trial and set the agent at the coordinates (1000, 1000). We assume that the agent moves in 2D square lattices. The field size is defined as 2000 × 2000. Periodic boundaries are assumed. In this algorithm, the agent memorizes the current patch as a visited patch when it leaves that place. The agent located at the coordinates (*x*, *y*) scans four discrete patches, (*x *+ 1, *y*), (*x *− 1, *y*), (*x*, *y *− 1) and (*x*, *y *+ 1), and selects one unvisited patch from among these four. If all of the above patches have been visited, then the agent cannot update the current position, which results in completion of that trial. We define the lifespans of trials as the lengths of time until trials are over. Actually, we found that some trials in 2D switching SAWs of recently visited locations finished before 1000 time steps. The agent can memorize each visited patch for a certain number of time steps. In this study, we name this value as the parameter *Memory_length* and set *Memory_length* = 10 as a default value. A visited patch turns to an unvisited patch after *Memory_length* time steps have passed since the agent left that patch; in other words, the agent forgets that position after a while.

**Figure 1.**
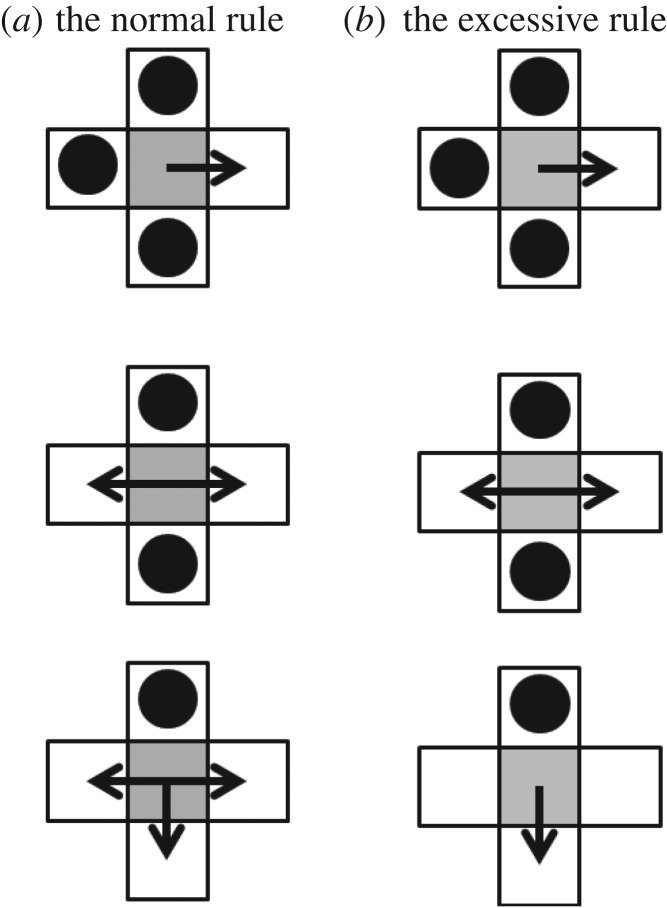
Schematic illustrations of reaction rules. (*a*) The normal reaction, (*b*) the excessive reaction. Previously visited patches are marked by black dots. Grey patches indicate the current positions of the agent. The agent is allowed to move in the directions shown as black arrows.

In the switching SAWs of recently visited locations, the agent uses two different reactions to avoid visited patches. One is the normal reaction. That is, the agent located at the coordinates (*x*, *y*) randomly selects one unvisited patch from among four patches: (*x *+ 1, *y*), (*x *− 1, *y*), (*x*, *y *+ 1) and (*x*, *y *− 1). The other one is the excessive reaction in which the agent moves in the opposite direction of visited patches when only one patch is a visited patch among four discrete patches: (*x *+ 1, *y*), (*x *− 1, *y*), (*x*, *y *− 1) and (*x*, *y *+ 1) (figure 1). For example, if the coordinate (*x *+ 1, *y*) is visited whereas others are not visited by the agent, then the agent updates its position from (*x*, *y*) to (*x *− 1, *y*).

At each time step, the agent adopts reaction rules in the following manner:

$$P_{\mathrm{normal}}=1-r,P_{\mathrm{excessive}}=r,P_{\mathrm{normal}}+P_{\mathrm{excessive}}=1.00,$$

where *P*_normal_ and *P*_excessive_ represent the probabilities of the normal reaction/excessive reaction at each time step, respectively.

The parameter *r* represents a probability and implies the excessive reaction sensitivity of the agent.

In the switching SAW of recently visited locations, if the agent experiences the following event, the agent changes the *r* in the following manner:

If |VS| = 3 and |UVS| = 1

Then,

$$\begin{array}{l} \;\;\text{if}\;r=0.00,\\ \;\;r\in [0.50,\;0.90]=\{r|0.50\leq r\leq 0.90\}\\ \text{else if}\;r>0.00\\ \;\;r=0.00, \end{array}$$

where VS and UVS represent the set of visited/unvisited patches, respectively, and satisfy the following equations:

VS∪UVS=S, VS⊆S, UVS⊆S,

where *S *= {(*x *+ 1, *y*), (*x *− 1, *y*), (*x*, *y *+ 1), (*x*, *y *− 1)}.

When the current position of the agent is (*x*, *y*).

Here, |*A*| represents the number of elements belonging to set *A*.

For example, if the agent located at the coordinates (*x*, *y*) scans four discrete patches (*x *+ 1, *y*), (*x *− 1, *y*), (*x*, *y *− 1) and (*x*, *y *+ 1) and detects that the coordinates (*x *+ 1, *y*) are an unvisited patch while the other three patches have been visited, the agent changes the *r*.

Note that, after changing the parameter *r*, the agent uses the changed parameter *r* until the above event occurs again.

In each trial, *r* is set to 0.00 as the initial condition.

Thus, if three visited patches surround the agent, the agent recognizes that the surrounding spaces have already been searched by it. To this end, the agent tends to move in the opposite direction from visited patches by changing the *r*. However, after changing the *r*, if the agent is again surrounded by any three visited patches, the agent returns the *r* to the default value (0.00). Therefore, the agent attempts to re-estimate the spaces that it has visited.

## 3. Results

**Figure 2.**
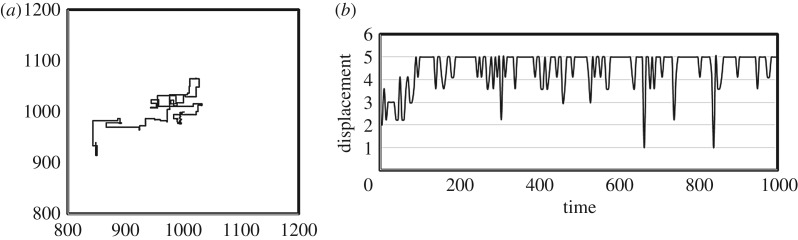
An example of trajectories and distances travelled every five time steps. (*a*) An example of trajectories of the agent from one trial. (*b*) An example of trajectories of the agent for every five time steps from one trial.

Figure 2*a* presents an example of the agent's trajectories obtained from one trial. According to figure 2*b*, which illustrates the distance travelled every five steps, the agent sometimes searches restricted areas while travelling across wide areas. The agent appears to produce both short steps and long steps.

In the simple random walk analysis, it is known that mean squared distance and time step are related by the following relation [17]:

$$\langle R^{2}\rangle ∼t^{2H}.$$

**Figure 3.**
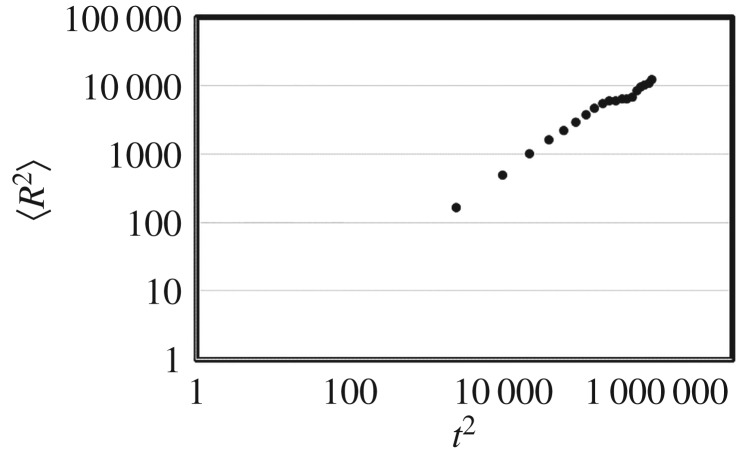
The mean squared distance and the time step obtained by the switching self-avoiding walk. Averaged *R*^2^ was obtained from 100 trials at each discrete time step. Data were obtained every 50 time steps.

Parameter *H* is determined depending on the algorithm (*H *> 1/2 in Lévy walk (super-diffusion), *H *= 1/2 in correlated random walk at more than 10^2^ time steps or Brownian walk (diffusion)). The diffusiveness of the search walks is one interesting feature to study to analyse search efficiency [18]. Note that *H *> 1/2 in pure 2D SAWs [19]. Figure 3 shows the mean squared distance and the time step obtained by our algorithm (averaged *R*^2^ was obtained from 100 trials at each discrete time step; data were obtained every 50 time steps). The fit for parameter *H* according to the model above was *H *∼ 0.71, indicating that super-diffusion was achieved (*R*^2^ = 0.99).

**Figure 4.**
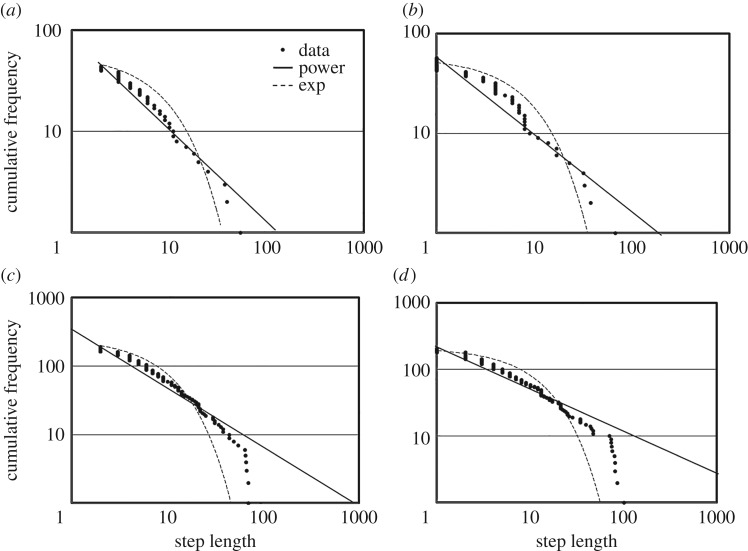
An example of step lengths of the agent. (*a*) The relationship between *x*-axis step lengths and those cumulative distributions obtained from one trial. (*b*) The relationship between *y*-axis step lengths and those cumulative distributions obtained from one trial. (*c*) The relationship between *x*-axis step lengths and those cumulative distributions obtained from five trials. (*d*) The relationship between *y*-axis step lengths and those cumulative distributions obtained from five trials. ‘Data' indicates step length data calculated from that trial. ‘Power’ indicates best-fit power-law distributions. ‘Exp' indicates best-fit exponential law distributions. Note that these data were obtained only from trials whose lifespans were more than 500 time steps.

Figure 4*a,b* represents the relationship between step length and its cumulative frequency in each direction. Note that it is theoretically impossible for the switching SAW model of recently visited locations to generate heavy tails because the model is Markovian. However, power-law tailed distributions were achieved across some ranges in both directions (*x*-direction: *n *= 44, *μ *= 1.89, weights of power law against exponential law = 0.90, goodness of fit test (GOF): *G*^2^ = 4.20, d.f.* *= 2, *p *= 0.12; *y*-direction: *n *= 57, *μ *= 1.74, weights of power law against exponential law = 1.00, GOF: *G*^2^= 2.14, d.f*. *= 3, *p *= 0.54). Here, step lengths were defined as distances along each direction between two consecutive turns [20]. Note that these data were obtained from one trial whose lifespan was more than 500 time steps because some trials were finished before any reaction changes. Here, we calculated the maximum-likelihood estimate of *µ* or *λ*, which is considered to give the most accurate results [21,22]. Note that a GOF for the data was also conducted to ensure that the preferred model is not just the best out of a bad lot.

Owing to the limited amount of plotted data from one trial, we summed data obtained from five trials whose lifespans were more than 500 time steps. Figure 4*c,d* indicates the results. As shown in figure 4, power-law tailed step lengths were achieved across some ranges (*x*-direction: *n *= 194, *μ *= 1.82, weights of power law against exponential law = 1.00, GOF: *G*^2^ = 6.00, d.f*. *= 5, *p *= 0.31; *y*-direction: *n *= 205, *μ *= 1.60, weights of power law against = 1.00, GOF: *G*^2^ = 6.47, d.f*. *= 4, *p *= 0.17). Electronic supplementary material, figure S1 presents examples obtained from two different trials regarding the relationships between the time interval of return visits and their frequency. As you can see, the agent in our model sometimes returns to previously visited sites even though it basically avoids recently visited ones. The agent in electronic supplementary material, figure S1A appears to return to recently visited sites whereas the agent in electronic supplementary material, figure S1B shows different behaviours; that is, it returns to previously visited sites after a while.

**Figure 5.**
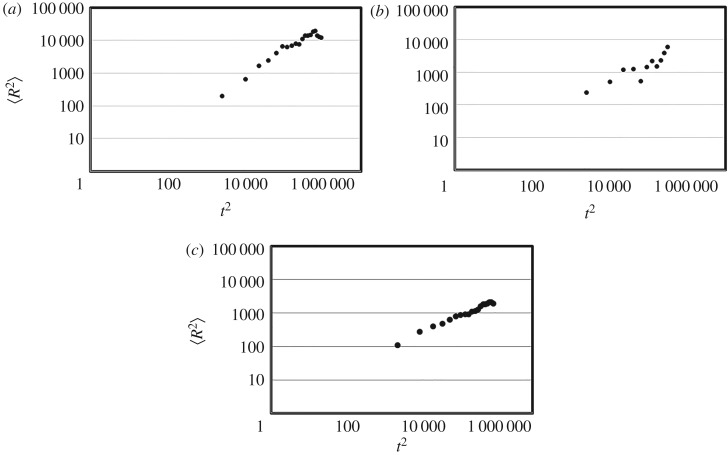
The mean squared distance and the time step obtained by replacing the parameter *Memory_length* = 10 with 20 (*a*), 10 with 50 (*b*), and by prohibiting the excessive reaction (*c*). Averaged *R*^2^ was obtained from 100 trials at each discrete time step. Data were obtained every 50 time steps.

Next, we checked how the parameter *Memory_length* influenced the agent's behaviours. Figure 5*a,b* illustrates the mean squared distance and the time step obtained by replacing *Memory_length *= 10 with 20 and 50, respectively (averaged *R*^2^ was obtained from 100 trials at each discrete time step; data were obtained every 50 time steps). The fit for parameter *H* according to the model above was *H *∼ 0.73 (*Memory_length *= 20) and *H *∼ 0.56 (*Memory_length *= 50), indicating that super-diffusion disappeared when that parameter became larger (*R*^2^ = 0.95 and 0.80, respectively). These findings indicate that the memory capacity of the agent does not need to be high.

Lastly, we examined whether super-diffusion was maintained if the agent only obeyed the normal reaction. Figure 5*c* illustrates the mean squared distance and the time step in this case (averaged *R*^2^ was obtained from 100 trials at each discrete time step; data were obtained every 50 time steps). The fit for parameter *H* according to the model above was *H *= 0.48–0.50, indicating that super-diffusion was not achieved (*R*^2^ = 0.99). Therefore, it appears that simple SAWs of recently visited locations with memory loss cannot achieve effective searching.

## 4. Discussion

Our results suggest that agents in the switching SAW of recently visited locations can produce power-law tailed movements across some ranges. In the above model, the agent changes its reaction to visited patches, depending on its experiences. If the agent is surrounded by visited patches, it recognizes that it has searched local areas and then tends to move in the opposite direction to explore unsearched areas. The agent subjectively estimates its global visited areas using local limited information because its memory disappears after a while. The agent estimates the directions that have already been searched. However, when the agent is again surrounded by visited patches, it temporarily searches surrounding areas by returning its reaction rules to the default rule. By doing so, the agent re-estimates the direction of unsearched areas.

In addition, hierarchical random walks obtained from changing the parameter *r* might enable the agent to produce reliable power-law distributions over some ranges. The agent in our model can produce one to many correspondences to one event (surrounded by visited patches). In that sense, the agent might change its behaviour spontaneously or in intrinsic ways [23–25]. The important point is that the agent can effectively search wide areas because we do not allow it to cross the areas that it recently searched. Therefore, the agent might go to new areas effectively if it is located around regions with a dearth of targets. However, the agent can sometimes return to visited sites after a while if it sets the parameter *r* to a smaller value. That is, the agent occasionally stays around or returns to visited regions, which might provide an opportunity for it to relocate regions rich in targets.

The agent in our model is not allowed to detect its global space-map. Therefore, it must predict its global space-map based on its recent memory. This assumption is plausible because foragers do not need to exploit high-level spatial cognition [26,27]. The use of local information for estimating migration patterns might produce many possibilities with respect to limited cues.

## Supplementary Material

Supporting Figures

## Data accessibility

All data are presented in the manuscript and the electronic supplementary material.
