# Supplementary material for: Optimal random search using limited spatial memory
Source: R Soc Open Sci. 2018 Mar 7;5(3):171057. doi: 10.1098/rsos.171057 (PMC5882669; doi:10.1098/rsos.171057)
Supplement: Supporting Figures [file rsos171057supp1.docx]

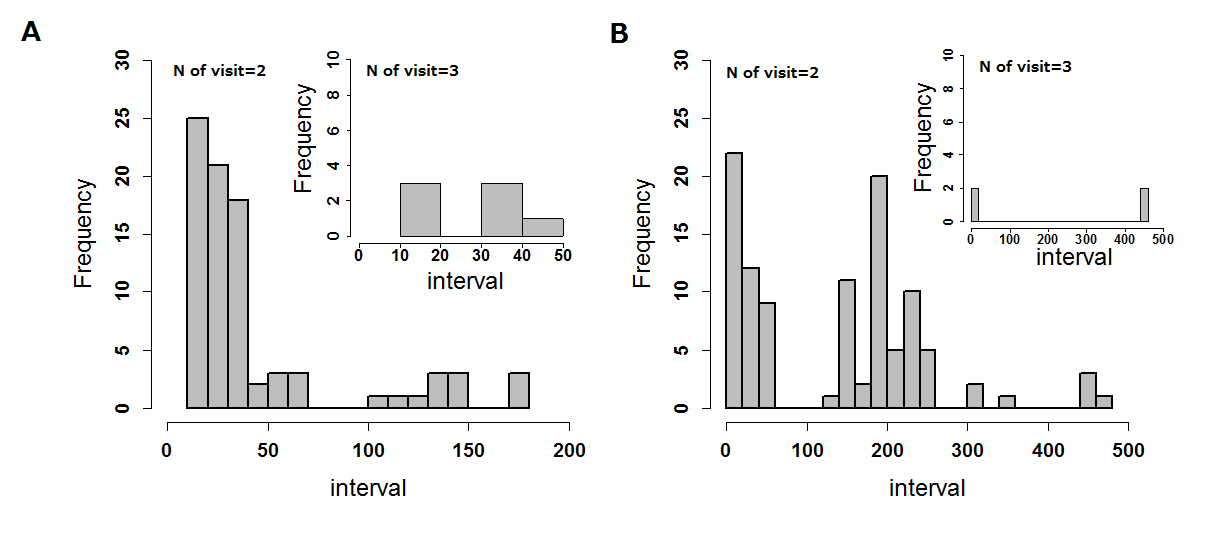


**Figure S1**. Relationships between the time interval of return visits and their frequency. Both A and B show the results from two different trials (lifespan = 1,000). *N* of visits = 2 indicates that the agent returns to those sites only once. *N* of visits = 3 indicates that the agent returns to those sites two times. Note that the results of *N* of visits = 1 (the agent does not return to those sites after leaving those sites) are not shown.
